# Nox4 is a Target for Tuberin Deficiency Syndrome

**DOI:** 10.1038/s41598-018-21838-4

**Published:** 2018-02-28

**Authors:** Qian Shi, Suryavathi Viswanadhapalli, William E. Friedrichs, Chakradhar Velagapudi, Cédric Szyndralewiez, Shweta Bansal, Manzoor A. Bhat, Goutam Ghosh Choudhury, Hanna E. Abboud

**Affiliations:** 1 Departments of Medicine/Division of Nephrology, University of Texas Health San Antonio, San Antonio, Texas USA; 2 Department of Cellular and Integrative Physiology, University of Texas Health San Antonio, San Antonio, Texas USA; 3 VA Biomedical Laboratory Research, South Texas Veterans Health Care System, San Antonio, Texas USA; 4 Geriatric Research, Education and Clinical Center, South Texas Veterans Health Care System, San Antonio, Texas USA; 5 Genkyotex SA, Geneva, Switzerland

## Abstract

The mechanism by which *TSC2* inactivation or deficiency contributes to the pathology of tuberous sclerosis complex (TSC) is not fully clear. We show that renal angiomyolipomas from TSC patients and kidney cortex from *Tsc2*+/− mice exhibit elevated levels of reactive oxygen species (ROS). Downregulation of tuberin (protein encoded by *TSC2* gene) in renal proximal tubular epithelial cells significantly increased ROS concomitant with enhanced Nox4. Similarly, we found elevated levels of Nox4 in the renal cortex of *Tsc2*+/− mice and in the renal angiomyolipomas from TSC patients. Tuberin deficiency is associated with activation of mTORC1. Rapamycin, shRNAs targeting raptor, or inhibition of S6 kinase significantly inhibited the expression of Nox4, resulting in attenuation of production of ROS in tuberin-downregulated proximal tubular epithelial cells. In contrast, activation of mTORC1 increased Nox4 and ROS. These results indicate that Nox4 may be a potential target for tuberin-deficiency-derived diseases. Using a xenograft model from tuberin-null tubular cells in nude mice, both anti-sense Nox4 and GKT137831, a specific inhibitor of Nox1/4, significantly inhibited the tumor growth. Thus, our results demonstrate the presence of an antagonistic relationship between tuberin and Nox4 to drive oncogenesis in the tuberin deficiency syndrome and identify Nox4 as a target to develop a therapy for TSC.

## Introduction

Tuberous Sclerosis Complex (TSC) is an autosomal dominant disorder syndrome characterized by the formation of tumors in various organs, such as brain, heart, kidneys, eyes, and skin. The main cause of TSC is the mutation(s) in either *TSC1* or *TSC2* genes, which encode hamartin (130 kDa) or tuberin (200 kDa), respectively. The third core subunit of this complex, TBC1D7, stabilizes the TSC complex^1^. The patients with TSC very likely develop some forms of kidney disease during their lifetimes, such as renal cysts, renal angiomyolipoma and renal cell carcinoma (RCC)^2–4^. Renal angiomyolipomas are usually the greatest concern in TSC patients with a frequency of about 80%, as the bleeding within angiomyolipomas is often life-threatening^2^. Renal cysts are often small with a frequency of 14–32%^5–7^, but can also lead to increased blood pressure, which may require dialysis or transplantation because of kidney impairment or kidney failure. The incidence of renal cell carcinoma (RCC) is normally underestimated due to the lower awareness and surveillance^8^, but often occurs in children and young adults^9,10^.

Mouse models of TSC show that homozygous *Tsc* null mice are embryonic lethal, while the heterozygous mice are viable and fertile. All *Tsc2*^+/−^ mice develop multiple bilateral renal cystadenomas at the age of 12–15 months, along with liver hemangiomas. Less than 10% of these mice develop renal cell carcinoma. TSC2 loss of heterozygosity (LOH) is common in all renal cystadenomas and renal carcinoma^11,12^. Eker rat, which carries a germ-line spontaneous mutation in *Tsc2* gene, is another rodent model for TSC disease^13^.

Reactive oxygen species (ROS) are known to increase significantly during the pathogenesis of various kidney diseases and in many cancers^14^. In addition to genetic deficiency as described above, tuberin can also be inactivated by phosphorylation at specific residues. We have previously shown that in kidney cortex of diabetic rodents, tuberin is inactivated by phosphorylation at Thr^1462^ together with an increase in ROS levels^15^. However, the mechanism by which tuberin deficiency/inactivation increases ROS levels is unknown. The understanding of the mechanism will help us identify the new therapeutic option for treating TSC patients.

Along with mitochondria, ROS are predominantly produced by the NADPH oxidase (NOX) complex in the cell membranes^16^. Nox enzymes catalyze the NADPH-dependent reduction of oxygen to form superoxide, which can dismutate to form hydrogen peroxide (H_2_O_2_). To date, seven members are identified in phagocytic and non-phagocytic cells, named Nox1–5, and Duox (dual Nox) 1–2. All subunits of NADPH oxidases, including p22^*phox*^, p47^*phox*^ and p67^*phox*^, as well as the isoforms Nox1, 2 and 4, are located in the various renal cells^17^. The exact function of each NADPH oxidase in regulating physiological and pathophysiological processes in the kidney is unclear. Nox4, first named as Renox, is the predominant NADPH oxidase expressed in the kidney, including vasculature, glomeruli, podocytes and mesangial cells^16,18,19^.

In the present study, we show increased levels of ROS in the renal angiomyolipomas of patients with TSC and the kidney cortex of *Tsc2* heterozygous mice. Using renal proximal tubular epithelial cells, we demonstrate that increased expression of Nox4 causes augmented ROS in a mTORC1-dependent manner. Inhibition of Nox4 by anti-sense oligonucleotide blocked the tumor formation in a mouse xenograft model of tuberin-deficient cells. Finally, we show the efficacy of a Nox1/4-specific inhibitor in ameliorating the tumor growth in mice.

## Results

### Tuberin deficiency leads to increase in ROS levels ***in vivo*** and ***in vitro***

**Figure 1:**
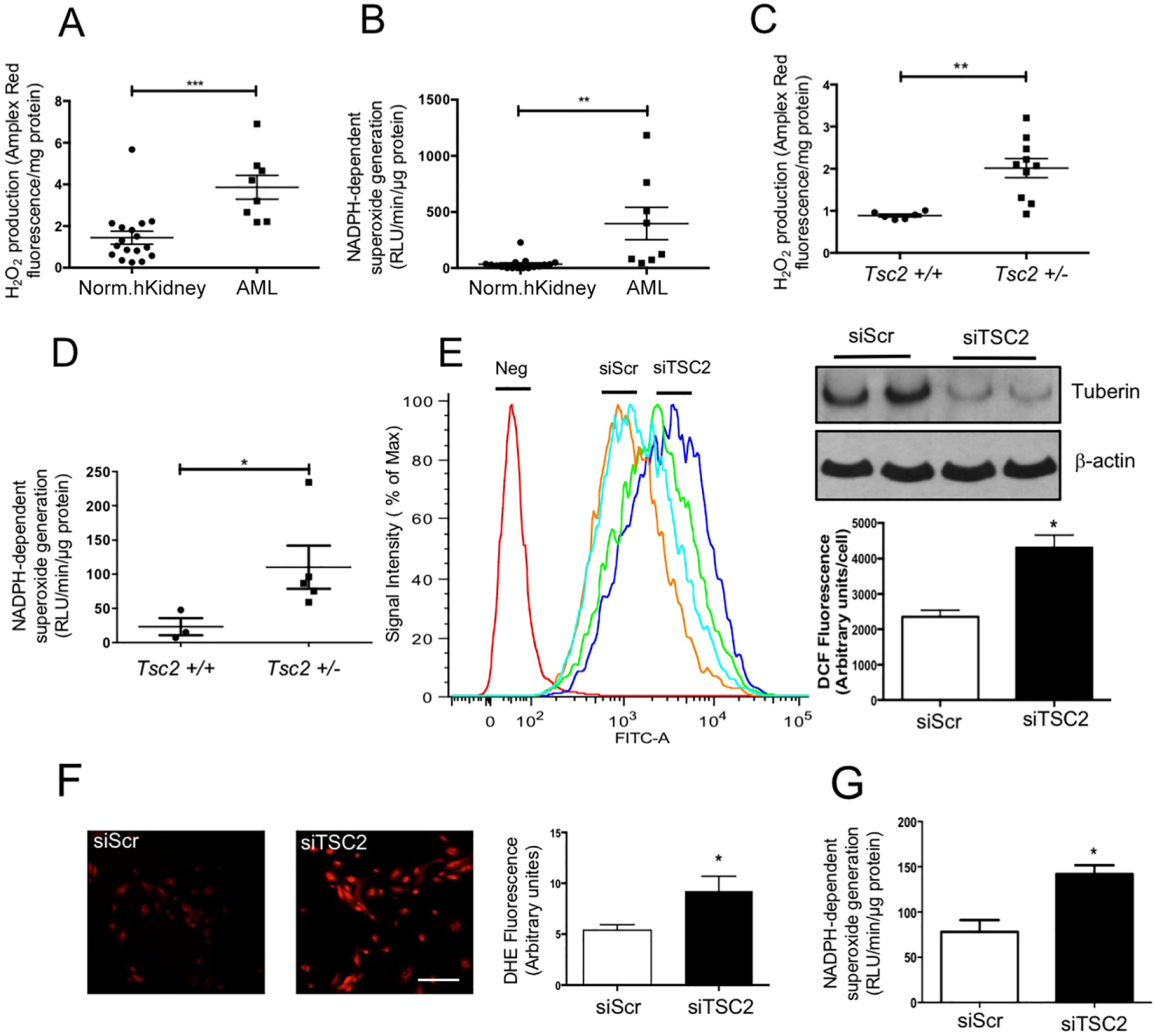
ROS levels are increased in human angiomyolipomas and in *Tsc2*+/− mouse model. (**A**) H_2_O_2_ levels and (**B**) Superoxide production in renal angiomyolipoma samples (AML) from TSC patients (n = 8) and normal kidney biopsy (*n* = 17) were shown, measured by Amplex red assay and lucigenin chemiluminescence, respectively. (**C**) H_2_O_2_ levels and (**D**) Superoxide production in control (*n* = 6) and *Tsc2*+/− mouse (*n* = 10) kidneys was measured by Amplex red and lucigenin chemiluminescence assays, respectively. (**E**) ROS levels were measured by flow cytometry with DCF dye in proximal tubular epithelial cells with siScramble (orange and light blue) or si*TSC2* (green and dark blue) (left panel). The top right panel shows the western blotting for tuberin protein levels in these cells. The bottom right panel showed the quantification of the data for DCF dye measurement. (**F**) ROS levels were measured with DHE dyes, scale bar: 200 μm and the intensity of staining per cells were quantified (right panel). Mean ± SE of 3 independent experiment, *p < 0.05 vs siScr; (**G**) Superoxide production was measured in siScramble or si*TSC2* transfected cells by lucigenin chemiluminescence. The data are presented as the mean ± S.E of 3 repeats. *p < 0.05; **p < 0.01; ***p < 0.001 in comparison with normal human kidney (**A**,**B**), or control mice (**C**,**D**), or cells receiving scramble siRNA (**E**–**G**).

Increased levels of ROS are commonly found in many cancer cells^20–22^. However, very limited data are available about renal ROS levels in TSC patients. We determined the levels of ROS in renal angiomyolipoma samples from the TSC patients by measuring the Amplex Red fluorescence, which detects the levels of hydrogen peroxide (H_2_O_2_). As shown in Fig. 1A, the levels of H_2_O_2_ were significantly higher in the angiomyolipomas as compared to that in the normal kidney tissues. Since NADPH oxidases are known to generate ROS in the kidney, we performed lucigenin chemiluminescence assay to measure the enzymatic activity of Nox proteins, which generate superoxide^23^. Nox enzymatic activity was significantly higher in the renal angiomyolipomas than that in normal human kidneys (Fig. 1B). Next, similar measurements were performed in the kidney cortex of *Tsc2*^+/−^ mice and control littermates at the age of 12–14 months. Both H_2_O_2_ levels and Nox activity were significantly higher in the kidneys of *Tsc2*^+/−^ mice than in the control mice kidneys (Fig. 1C,D). To further examine the mechanism how tuberin deficiency leads to increase in ROS levels, we used primary human renal proximal tubular epithelial cells, which represent the cells for renal tumors associated with tuberin deficiency^24^. *TSC2* siRNA were used to specifically knock down expression of tuberin in the proximal tubular epithelial cells (Fig. 1E, top right panel). We measured ROS generation in cells by using oxidant-sensitive H_2_DCFDA dye, which is a cell-permeant indicator for general ROS, mainly H_2_O_2_^20^. The measurement of mean DCFDA fluorescence showed that ROS levels were significantly increased in *TSC2* knockdown cells (Fig. 1E). To confirm this observation, we used DHE, another cell-permeable ROS-sensitive probe (mainly superoxide)^20^, to measure the total levels of ROS (Fig. 1F). Both *in vivo* detection of ROS in Fig. 1E,F show that siRNAs against tuberin significantly increased the levels of ROS in the human proximal tubular epithelial cells. Similarly, Nox enzymatic activities were also elevated in the cells transfected with siTSC2 (Fig. 1G). Together, these data show the presence of elevated levels of ROS in renal angiomyolipomas from patients with TSC and in the kidney tissue from tuberin deficient mice. Furthermore, our results demonstrate increased production of ROS *in vitro* in siRNA-treated proximal tubular epithelial cells.

### Tuberin regulates expression of Nox4 NADPH oxidase

**Figure 2:**
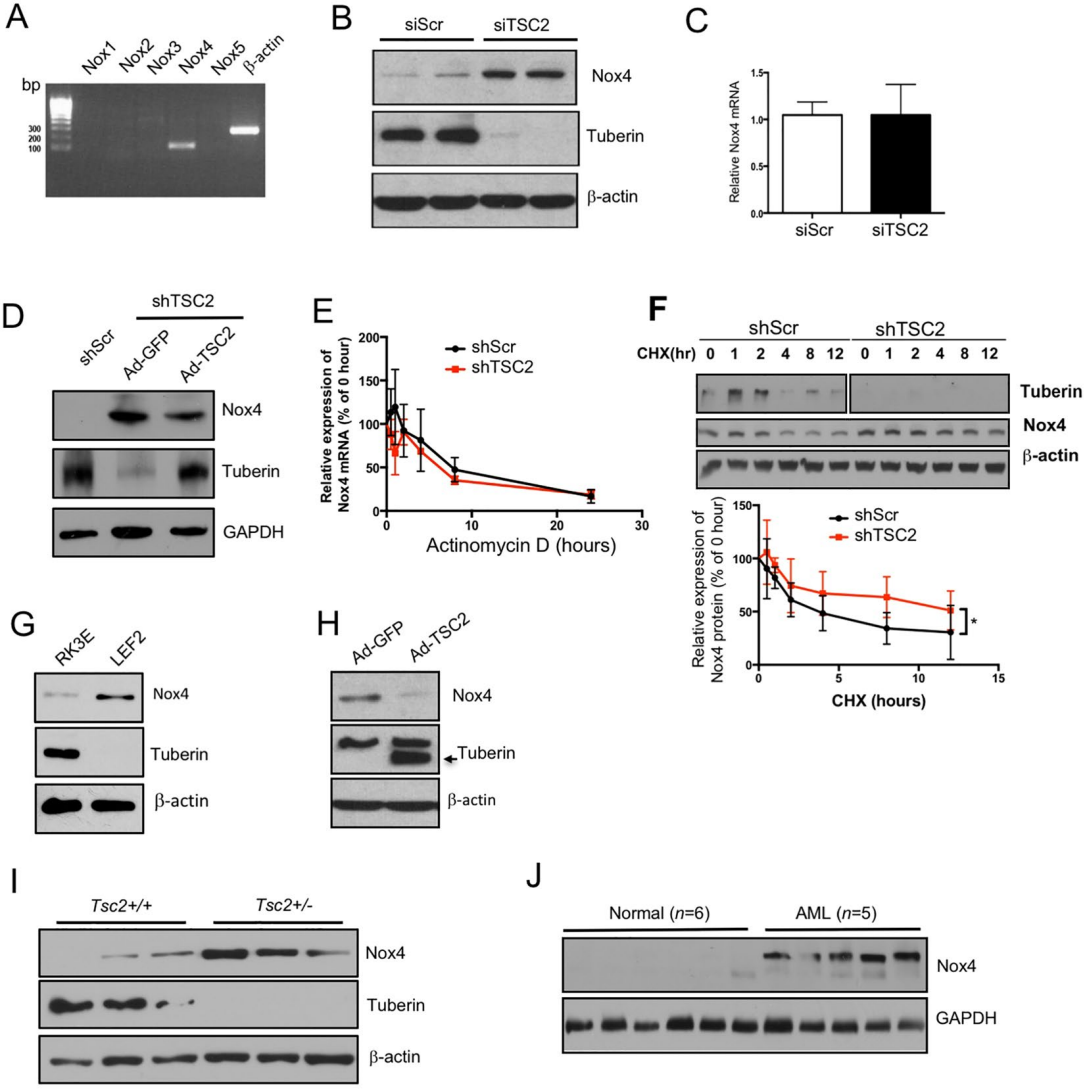
Tuberin regulates Nox4 protein expression. (**A**) RT-PCR analysis of the Nox isoforms in human proximal tubular epithelial cells with primers listed in Supplementary Table 1. (**B**) Nox4 protein levels were determined by western blotting in human proximal tubular epithelial cells transfected with Scramble or *TSC2* siRNA; (**C**) mRNA levels were measured by qRT-PCR in cells transfected with Scramble or *TSC2* siRNA; The data are presented as the mean ± S.E, in comparison with cells receiving scramble siRNA (*n* = 4). (**D**) Proximal tubular epithelial cells stably expressing *TSC2* shRNA were infected with adenoviruses expressing GFP or TSC2; tuberin and Nox4 protein levels were measured in these cells by western blotting. (**E**) Nox4 RNA stability was measured by qRT-PCR in Actinomycin D (5 μg/mL)-treated shTSC2 cells. (**F**) Protein stability of Nox4 was measured by western blotting using cycloheximide (CHX) (100 μg/mL)-treated shTSC2 cells for indicated times. Lower panel showed the percentage changes of protein during the CHX treatment; (**G**) Nox4 and tuberin protein levels were measured in RK3E and LEF2 cells by western blotting; (**H**) Nox4 and tuberin protein levels were measured in LEF2 cells infected with adenoviruses containing GFP or TSC2; (**I**) Nox4 and tuberin protein levels were measured in control and *Tsc2*^+/−^ mouse kidney cortices; (**J**) Nox4 protein levels were measured in renal angiomyolipoma samples from TSC patients and normal human kidney biopsies. In all western blotting assays, β-actin or GAPDH serves as loading controls.

Our results above demonstrate increased Nox activity in tuberin-deficient states. Seven isoforms of NADPH oxidases have been identified in humans. However, except Duox1/2 and Nox3, Nox1, Nox2, Nox4, and Nox5 are all detected in kidney tissue, and in various renal cells^17^. To determine the source that mainly contributes to the elevated ROS levels in renal proximal tubular cells, mRNAs for five Nox isoforms were analyzed by RT-PCR. As shown in Fig. 2A, only Nox4 mRNA was detected in renal tubular epithelial cells. To confirm the expression patterns of Nox isoforms, we performed quantitative RT-PCR using TaqMan probes (Supplementary Table 1). The results showed that Nox4 was indeed the most abundantly expressed NADPH oxidase in human proximal tubular epithelial cells (Supplementary Figure S1). Expression of Nox1 and 3 was barely detectable while Nox2 and Nox5 were expressed at 0.6% and 1.0% levels compared to Nox4. These results indicate that Nox4 is likely to be the major contributor to the overall level of ROS found in tuberin deficiency.

We next examined the effect of tuberin knockdown on the expression of Nox4. siRNAs against tuberin increased the expression of the Nox4 protein (Fig. 2B). Surprisingly, the mRNA levels of Nox4 were not altered in TSC2 siRNA transfected cells (Fig. 2C). To confirm the role of tuberin in Nox4 expression, we established proximal tubular epithelial cells deficient in tuberin by shRNA against tuberin. In these shTSC2 cells, expression of Nox4 was increased as compared to that in control cells (Fig. 2D, compare lane 2 with lane 1). Reconstitution of tuberin in these cells inhibited the expression of Nox4 (Fig. 2D, compare lane 3 with lane 2). These results demonstrate that Nox4 is regulated at the protein level. To confirm this mechanism that loss of tuberin regulates the protein expression of Nox4, we examined the stability of Nox4 mRNA in renal epithelial cells in the presence of actinomycin D. Actinomycin D showed similar Nox4 mRNA decay for both control and tuberin-deficient cells (Fig. 2E). These results indicate that tuberin does not regulate the expression of Nox4 at the mRNA level. Next, we determined the effect of cycloheximide on the protein abundance of Nox4. The results showed that cycloheximide significantly decreased the protein turnover in the shTSC2 cells (Fig. 2F), suggesting that tuberin regulates Nox4 expression at the level of protein. Similar to the regulation of Nox4 expression in shTSC2 cells, we confirmed this observation using the LEF2 cells derived from Eker rat tumors with tuberin-null phenotype^13,21^. The level of Nox4 protein was increased as compared to rat kidney epithelial cells, RK3E (Fig. 2G). In contrast, when tuberin expression was reconstituted in these cells, the expression of Nox4 was attenuated (Fig. 2H).

To determine whether the inverse correlation between tuberin and Nox4 observed in the proximal tubular epithelial cells exists *in vivo*, we examined the expression of Nox4 in the kidneys of *Tsc2*^+/−^ mice at one year of age. Consistent with the previous reports^11,12^, clear cystadenomas were identified in these kidneys. Analysis of the cystadenomas from these kidneys showed almost undetectable levels of tuberin with a concomitant increase in expression of Nox4 (Fig. 2I). Similarly, renal angiomyolipomas from patients with TSC exhibited significantly higher levels of Nox4, as compared to the normal kidneys (Fig. 2J). These results confirm the presence of a reciprocal correlation between the expression of tuberin and Nox4 in the kidney.

### mTOR complex 1 signaling regulates Nox4 protein expression in tuberin-deficient cells

**Figure 3:**
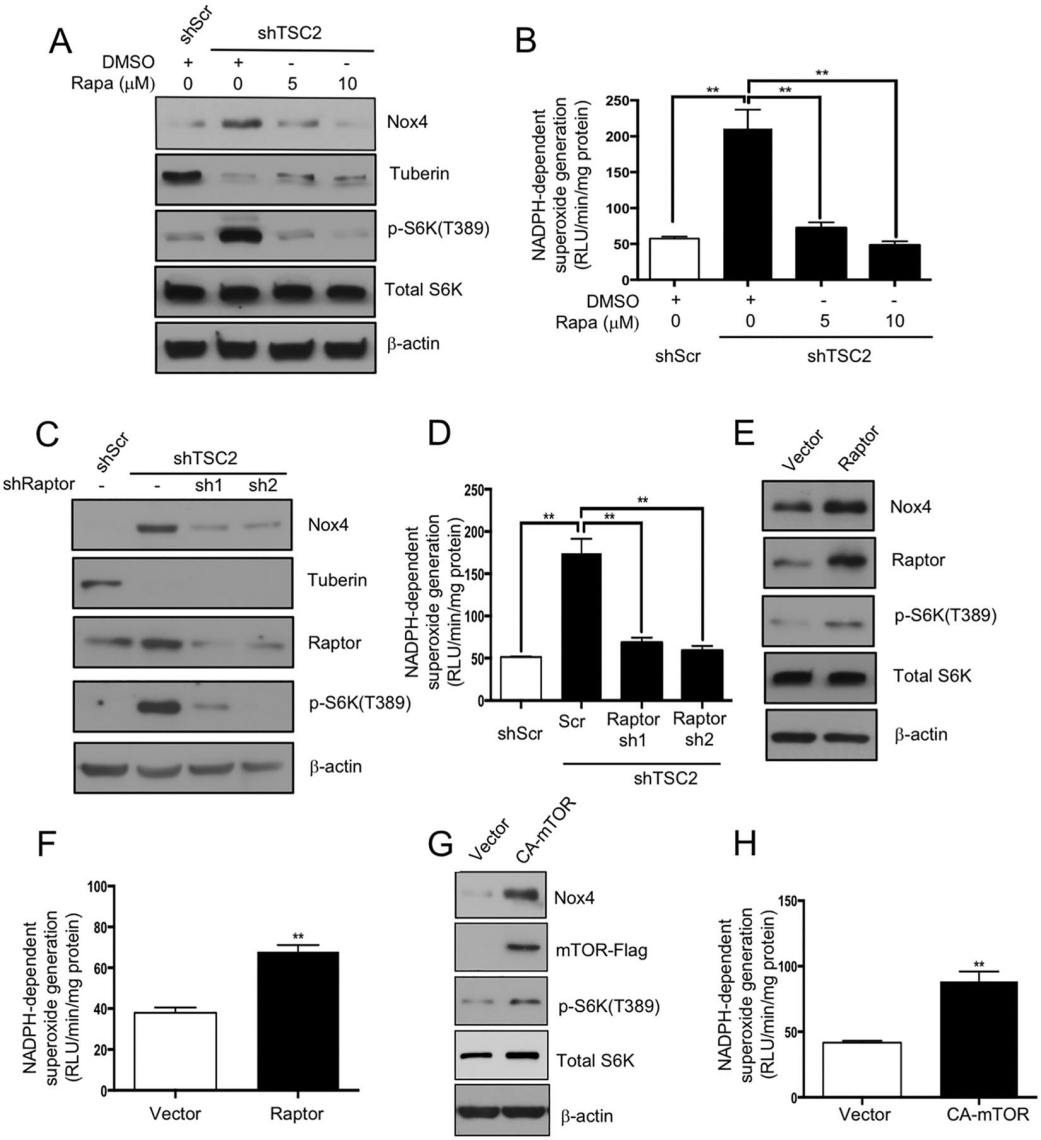
mTORC1 regulates Nox4 expression in tuberin-deficient cells. (**A** and **B**) ShScr and shTSC2 proximal tubular epithelial cells were incubated with rapamycin as indicated. The cell lysates were immunoblotted with indicated antibodies (panel A) and assayed for NADPH oxidase activity using lucigenin chemiluminescence assay (panel B). In panel B, mean ± SE of 5 independent experiments. **p < 0.01 between indicated groups. (**C** and **D**) ShScr and shTSC2 proximal tubular epithelial cells were transfected with raptor sh1 or raptor sh2. The cell lysates were immunoblotted with indicated antibodies (panel C) or assayed for NADPH oxidase activity using lucigenin chemiluminescence assay (panel D). In panel D, mean ± SE of 5 independent experiments. **p < 0.01 between indicated groups. (**E–H**) Proximal tubular epithelial cells were transfected with vector or raptor expression plasmids (panels E and F) or constitutively active (CA) mTOR expression plasmid (panels G and H). The cell lysates were immunoblotted with indicated antibodies (panels E and G) and assayed for NADPH oxidase activity using lucigenin chemiluminescence assay (panels F and H). In panels F and H, mean ± SE of 5 independent experiments. **p < 0.01 vs vector. In panels A,C,E and G, representative of 3 independent immunoblot experiments is shown.

mTORC1 is aberrantly activated in humans with TSC and in Eker rat^25^. Also, tuberin deficient murine embryonic fibroblasts showed increased mTORC1 activity^22^. Therefore, we investigated if tuberin regulates Nox4 expression through altered mTORC1 signaling pathway. Proximal tubular epithelial cells with downregulated expression of tuberin showed increased mTORC1 activity as judged by phosphorylation of its substrate S6 kinase. This increase in mTORC1 activity was associated with enhanced Nox4 expression (Fig. 3A, compare lane 2 with lane 1). Interestingly, incubation of tuberin-downregulated cells with rapamycin inhibited the mTORC1 activity concomitant with attenuation of expression of Nox4 (Fig. 3A, compare lanes 3 and 4 with lane 2). Similarly, rapamycin significantly inhibited the NADPH oxidase activity in tuberin-deficient cells (Fig. 3B). Downregulation of raptor, the specific and required component of mTORC1, by two independent shRNAs inhibited the expression of Nox4 and NADPH oxidase activity (Fig. 3C,D), thus confirming the effect of rapamycin. Furthermore, overexpression of raptor, which increased the mTORC1 activity, augmented the expression of Nox4 and its activity (Fig. 3,F). Moreover, expression of constitutively active mTORC1 increased the Nox4 expression and NADPH oxidase activity in the proximal tubular epithelial cells (Fig. 3G,H). These results demonstrate the involvement of mTORC1 in the expression of Nox4 responding to tuberin deficiency.

**Figure 4:**
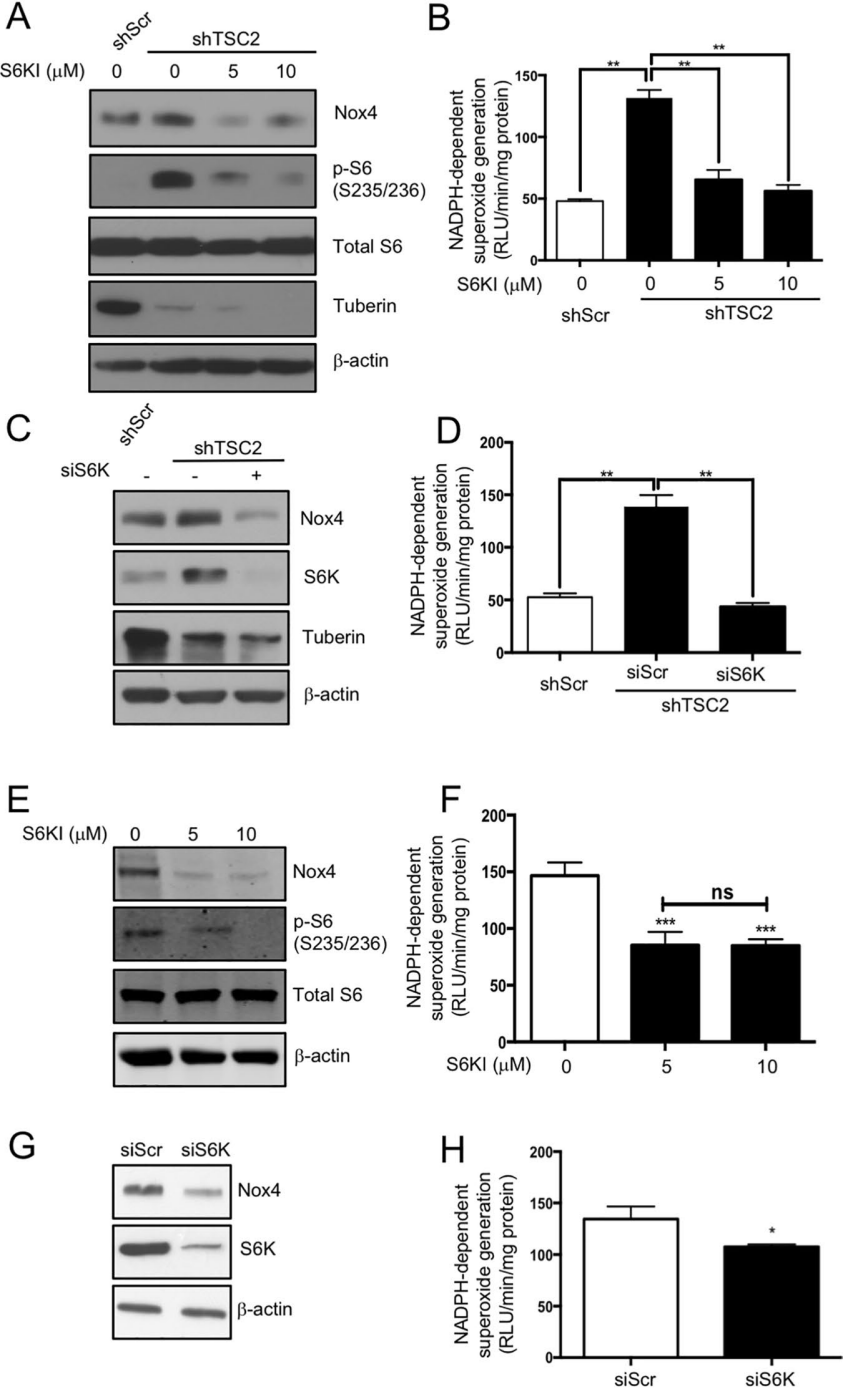
S6 kinase regulates Nox4 expression. (**A**,**B**) shTSC2 cells were incubated with S6 kinase inhibitor (S6KI) as indicated. The cell lysates were immunoblotted with indicated antibodies (**A**) or assayed for NADPH oxidase activity using lucigenin chemiluminescence assay (**B**). (**C**,**D**) shTSC2 cells were transfected with siRNAs against S6 kinase and the cell lysates were immunoblotted with indicated antibodies (**C**) and assayed for NADPH oxidase activity using lucigenin chemiluminescence assay (**D**). (**E**,**F**) LEF2 cells were treated with S6KI and the protein levels were examined by western blotting (**E**) and NADPH oxidase activity was measured by lucigenin chemiluminescence assay (**F**). (**G**,**H**) LEF2 cells were transfected with siRNAs against S6 kinase. Cell lysates were immunoblotted with indicated antibodies (**G**) and NADPH oxidase activities were measured by lucigenin chemiluminiscence assay (**H**). In panels A,C,E,G, representative of 3 independent immunoblot experiments is shown. In panels B,D,F and H, mean ± SE of 5 independent experiments. **p < 0.01, ***p < 0.001 between indicated groups.

Since S6 kinase is activated by mTORC1-mediated phosphorylation (Fig. 3A), we determined its requirement for Nox4 expression. Incubation of tuberin knockdown proximal tubular epithelial cells with an S6 kinase inhibitor (S6KI) blocked its kinase activity as judged by the phosphorylation of the small ribosomal protein s6 (Fig. 4A). Inhibition of S6 kinase activity resulted in decreased expression of Nox4 and the NADPH oxidase activity (Fig. 4A,B). Similarly, downregulation of S6 kinase using pools of siRNAs attenuated the Nox4 expression and the NADPH oxidase activity (Fig. 4C,D). To confirm these results, we used S6KI in LEF2 cells. S6KI inhibited the expression of Nox4, resulting in attenuation of NADPH oxidase activity (Fig. 4E,F). Similar results were found in LEF2 cells transfected with siRNAs against S6 kinase (Fig. 4G,H). These data for the first time show a role of mTORC1 target S6 kinase in regulation of Nox4 protein expression in tuberin deficiency.

**Figure 5:**
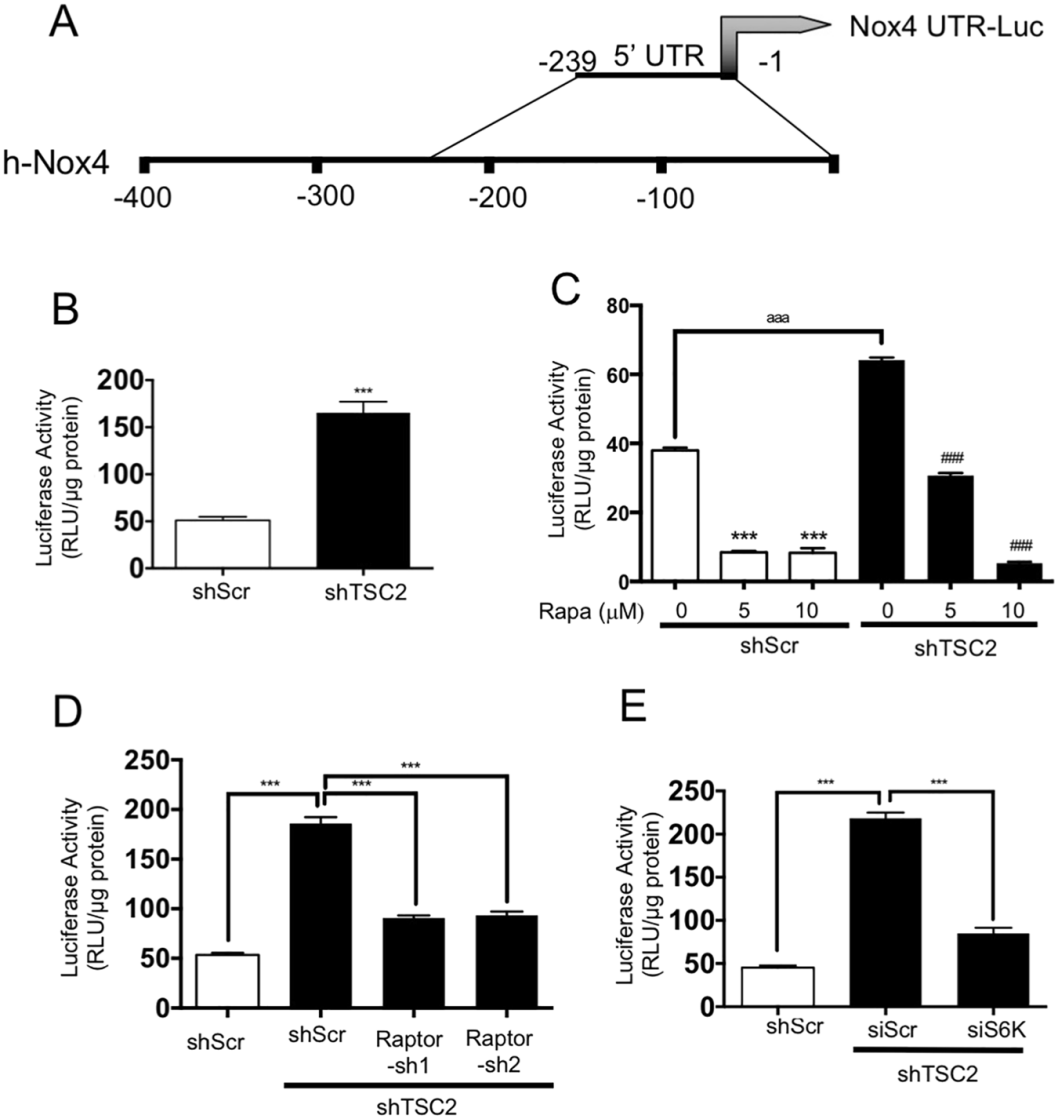
Tuberin deficiency regulates the expression of Nox4 at protein translational level. (**A**) Nox4 5′UTR (−239/−1) was derived from human Nox4 and sub-cloned into a vector upstream of the luciferase gene (Nox4 UTR-Luc) as described in the Methods. (**B**,**C**), Nox4 UTR-Luc was transfected into shTSC2 cells. In panels C, the transfected cells were treated with indicated concentrations of rapamycin. (**D**,**E**) shTSC2 cells were co-transfected with Nox4 UTR-Luc and Raptor shRNAs (panel D) or siRNAs against S6 kinase (panel E). Luciferase activity was determined in the cell lysates. The data shown are averages ± S.E.M. of RLU reading/μg protein from 3 individual experiments. **p < 0.01, ***p < 0.001.

To directly determine whether Nox4 is regulated at the protein level, we cloned the 5′ untranslated region (UTR) of Nox4 mRNA into a luciferase reporter vector (Nox4 UTR-Luc) (Fig. 5A). The luciferase activity derived from this vector represents the protein expression of Nox4. Tuberin deficient and control proximal tubular epithelial cells were transfected with the Nox4 UTR-Luc plasmid. The tuberin-deficient cells showed significantly increased luciferase activity suggesting that tuberin negatively regulates the Nox4 protein expression (Fig. 5B). mTOR has been shown to regulate the mRNA translation^26^. Also, our results above demonstrate that mTOR controls the Nox4 protein levels. Therefore, we examined the involvement of this kinase in Nox4 protein translation using this reporter construct in shTSC2 cells. Treatment of the reporter-transfected cells with rapamycin showed decreased luciferase activity (Fig. 5C). Similarly, shRNAs against raptor produced reduced luciferase activity in these cells (Fig. 5D). Furthermore, siRNAs against S6 kinase showed significantly reduced luciferase activity (Fig. 5E). These results indicate that tuberin regulates the expression of Nox4 at the protein level.

### Nox4 downregulation by anti-sense oligonucleotide suppresses the formation of tumor xenografts in nude mice

**Figure 6:**
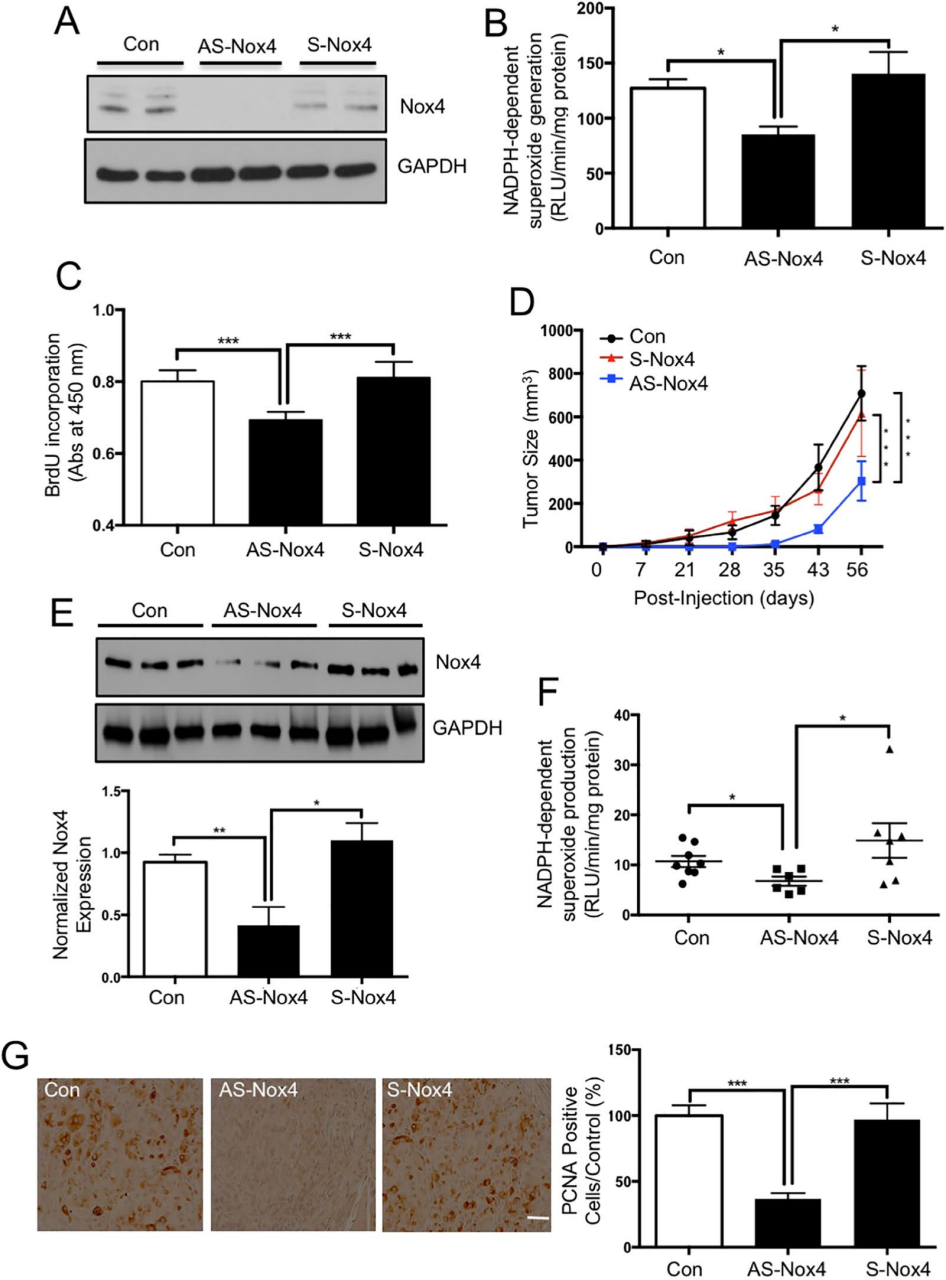
Knock down of Nox4 by antisense oligonucleotides significantly suppresses the formation of LEF2 tumor xenografts in nude mice. (**A**–**C**) Antisense (AS) and sense (S) oligonucleotides were transfected into LEF2 cells. 72 hours post-transfection, Nox4 protein levels by immunoblotting (panel A), superoxide production by NADPH oxidase activity (panel B) and cell proliferation by BrdU incorporation (panel C) were examined. (**D**) 6 weeks old nu/nu mice were inoculated with 3 × 10^6^ LEF2 cells subcutaneously. At the second day, PBS (Con.) or sense or anti-sense Nox4 oligonucleotides (45 mg/kg body weight/day, *n* = 8 for each group) were administered subcutaneously and the mice were monitored for 8 weeks. The tumor size was measured by caliper twice a week. ***p < 0.001 between indicated groups. (**E**) Nox4 protein levels were measured in extracts of tumors by western blotting. Each lane shows individual animal. Bottom panels show quantification of the band intensity. Mean ± SE of 8 mice in each group, **p < 0.01 between con. and As-Nox4 groups, and *p < 0.05 between As-Nox4 and S-Nox4 groups. (**F**) NADPH oxidase-dependent superoxide generation was determined in the extracts of tumors from the three groups described in panel D. *p < 0.05 between con. and AS-Nox4 group and between AS and S-Nox4; (**G**) Expression of PCNA was examined by immunohistochemistry in the control and tumor tissues (scale bar, 200 μm). The right panels show quantification of the stained cells. 10 random pictures for each animal were taken and mean values were used for each animal. Mean ± SE of 8 mice in each group were quantified. ***p < 0.001 vs. Con or S-Nox4.

Our results above show that increased levels of ROS due to augmented Nox4 expression in tuberin-deficient proximal tubular cells may drive the tumorigenic potential of these cells. Therefore, we sought to examine if inhibiting Nox4 would be a potential approach to suppress the formation of a tumor that originates from tuberin-deficient cells. We have previously used antisense oligonucleotide to block the function of Nox4 *in vivo*^27,28^. We transfected LEF2 *Tsc2* null cells with antisense oligonucleotide against Nox4; the expression of the Nox4 protein, NADPH oxidase activity and cell proliferation were measured after 72 hours (Fig. 6A,C). The attenuation of Nox4 expression, and subsequent decline in NADPH oxidase activity and cell proliferation suggested that inhibiting Nox4 protein translation could potentially reduce the ROS levels and proliferation of cells *in vitro*, indicating a significant role of Nox4 in inducing tumor cell growth. To determine the effect of the anti-sense Nox4 *in vivo*, we subcutaneously inoculated LEF2 (*Tsc2*^−/−^) cells to generate tumor xenografts in nude mice. At the second day, we administered anti-sense Nox4, sense Nox4 oligonucleotides or PBS and the tumor growth was monitored. As shown in Fig. 5D, tumor size was increased with time in the control groups. Treatment of mice with anti-sense Nox4 showed a significant reduction in tumor size as compared to sense-Nox4- or PBS-treated groups (Fig. 6D). To validate the specificity of the anti-sense Nox4, tumors were extracted at the end of the experiments and Nox4 expression and activity were measured. The tumors from the mice treated with anti-sense Nox4 showed reduced Nox4 expression and NADPH oxidase activity as compared to the PBS− or sense Nox4-treated groups (Fig. 6E,F). Consequently, the expression of PCNA, a marker of cell proliferation, was significantly attenuated in the tumors of anti-sense Nox4-treated animals (Fig. 6G). These data indicate that Nox4 contributes to the tumor growth derived from tuberin deficiency.

### Nox4-specific inhibitor GKT prevents tumor growth *in vivo*

**Figure 7:**
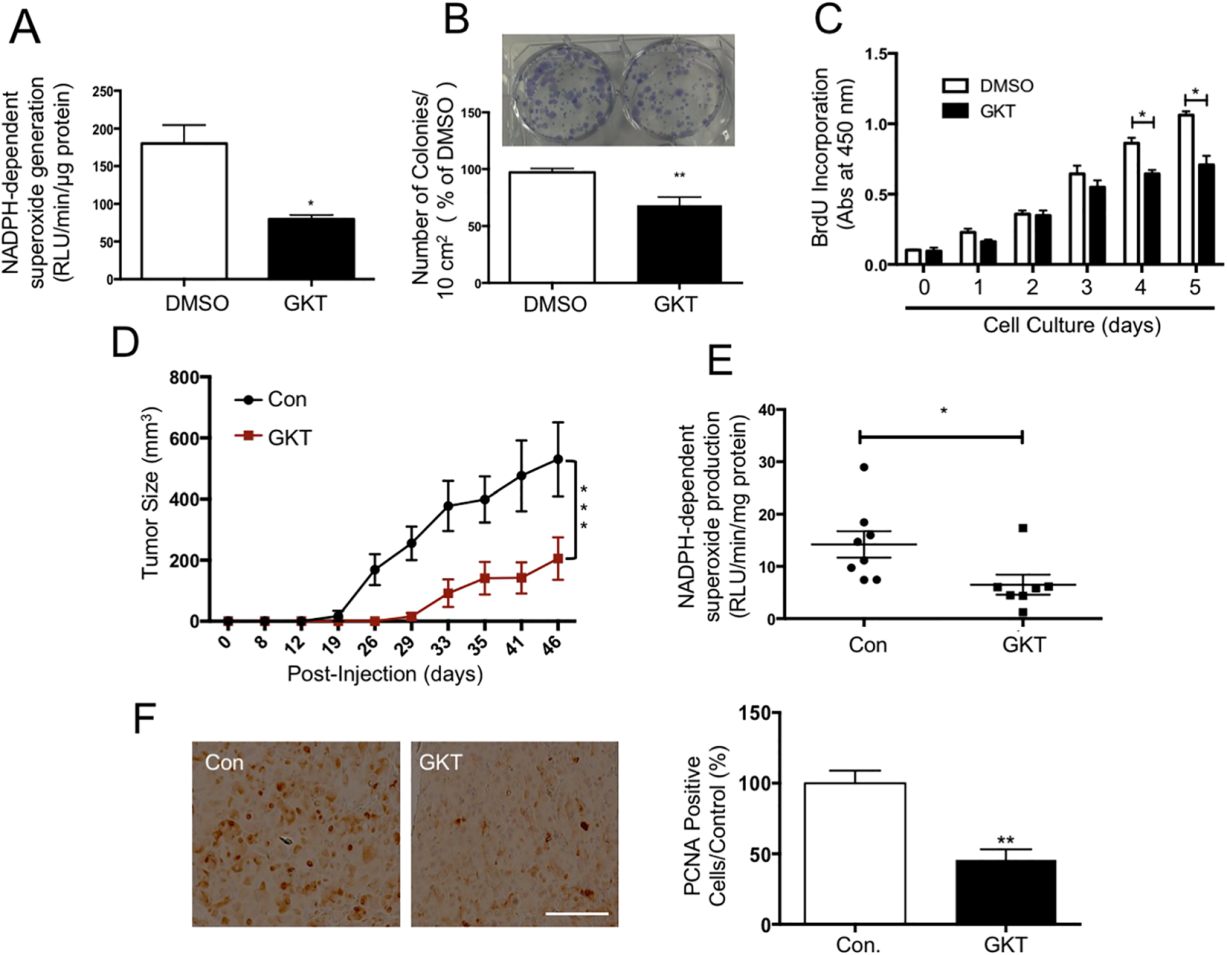
Nox1/4 inhibitor, GKT137831 (GKT) significantly suppresses the growth of tuberin-deficient LEF2 cells *in vitro* and LEF2 tumor xenografts in nude mice. (**A**) NADPH oxidase-dependent superoxide production was significantly suppressed in LEF2 cells treated with GKT for 24 hours, measured by lucigenin chemiluminescence. (**B**) Colony formation was inhibited by GKT in LEF2 cells; the images were taken and the measurement was performed at the 7^th^ day of treatment. **p < 0.01 between DMSO and GKT groups. (**C**) BrdU incorporation assay was performed in LEF2 cells with GKT for continuous 5 days, and absorbance was measured at 450 nm; (**A**–**C**) For A and B, mean ± SE of 3 experiments. For panel C, mean ± SE of 5 experiments; *p < 0.05, **p < 0.01 GKT *vs*. DMSO. (**D**) Normal diet (Con) and GKT diet (GKT) were fed to mice from the 2^nd^ day after inoculation of LEF2 cells. The tumor size was measured by caliper twice a week, during 7 weeks. 10 mice in each group were used. ***p < 0.001 *vs.* control. (**E**) NADPH oxidase-dependent superoxide generation was determined in the extracts of tumors from the two groups described in panel D (*n* = 8). *p < 0.05 *vs*. Control. (**F**) Expression of PCNA was examined by immunohistochemistry in the tumor samples from the control and GKT-treated mice (scale bar, 200 μm). The right panel shows quantification of the stained cells. 10 random pictures for each animal were taken and mean values were used for each animal. Mean ± SE of 8 mice **p < 0.01 *vs.* control.

A pharmacologic inhibitor of Nox1/4, GKT137831, has recently been developed and tested in preclinical models of various diseases^29,30^. Incubation of LEF2 cells with GKT showed a significant decrease in the NADPH oxidase activity, which resulted in marked inhibition of colony formation of these cells (Fig. 7A,B). Similarly, GKT inhibited proliferation of LEF2 cells starting at day 4 (Fig. 7C). The extent of inhibition of proliferation of LEF2 cells by Nox4 anti-sense oligo and GKT was similar (Figs 6C and 7C). Interestingly, rapamycin inhibited the expression of Nox4 concomitant with inhibition of the mTORC1 activity (Supplementary Figure S2A). Consequently, rapamycin significantly blocked the NADPH oxidase activity, which resulted in decreased colony formation of LEF2 cells (Supplementary Figure S2B and C). However, rapamycin significantly prevented the proliferation of LEF2 cells starting at day 2 (Supplementary Figure S2D). It should be noted that GKT did not alter the Nox4 protein expression (Supplementary Figure S3); but it blocked the activity of Nox4 (Fig. 7A). The comparison between rapamycin and GKT on cellular proliferation rates were also examined using human renal epithelial cells deficient in tuberin expression. Rapamycin reduced cellular proliferation starting at day-2; however, GKT only had effects starting at day-5 during the treatment (Supplementary Figure S4), which is in consistent with the results found in LEF2 cells. Since our results using anti-sense Nox4 inhibited the tumor growth of tuberin-deficient cells, we used this inhibitor in the LEF2 cell-derived tumor xenograft model described above. One-day post-inoculation of the LEF2 cells, the mice were fed normal chow or GKT-containing chow during the period of experiments. As shown in Fig. 7D, the GKT-fed mice showed a significant reduction in tumor sizes as compared to the normal chow-fed group. In chow-fed mice, the tumors were visible at 12 days, while in the GKT group the growth of the tumors was delayed (Fig. 7D). These results suggest that GKT prevented the rate of tumor growth. Also, the NADPH oxidase activity was significantly reduced in the tumors from the GKT-fed mice (Fig. 7E). Finally, the expression of the proliferation marker PCNA was markedly inhibited in the GKT-treated mice tumors (Fig. 7F). Together our data demonstrate the efficacy of a small molecule inhibitor of Nox4 in the tuberin deficiency-driven tumorigenesis.

## Discussion

**Figure 8:**
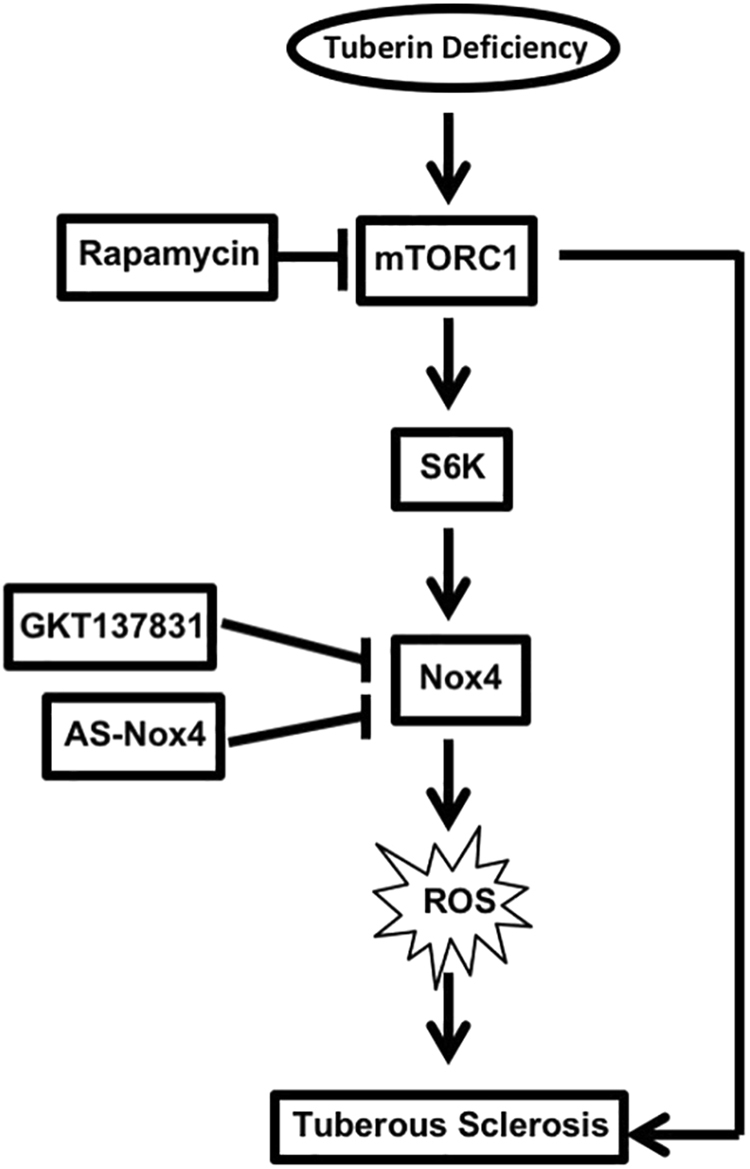
Schematic summarizing the results of the study.

Germline mutations in *TSC1* or *TSC2* genes cause benign tumors in multiple organs in patients with TSC. The disease is considered as a rare syndrome, as only one in 6,000 children is born with it. However, many cases may remain undiagnosed for years due to the relatively obscure nature of the disease. *TSC1/2* have been defined as tumor suppressor genes, as they are inactivated/deficient in many cancers, in which mTORC1 kinase is aberrantly activated^31,32^. Therefore, inhibition of this kinase has emerged as a potential therapeutic option for TSC patients. However, in patients with renal angiomyolipomas treated with mTOR inhibitor, the disease relapsed^33^. Therefore, the main purpose of current study is to identify further new mechanism that may drive the pathology of TSC, and identify new therapeutic option for treating TSC. In the present study, using renal proximal tubular cells, tuberin heterozygous mouse and patient-derived renal angiomyolipomas, we have identified the NADPH oxidase isoform Nox4 as the source of ROS due to tuberin deficiency (Fig. 8). We show that Nox4 is a downstream target of mTORC1/S6 kinase signaling, through the translational regulation of Nox4 mRNA. We demonstrate that anti-sense-mediated inhibition of Nox4 significantly reduced the tumor growth generated by tuberin-deficient cells in mice. Finally, we provide the first evidence for an alternative therapeutic option other than rapamycin using a Nox4 inhibitor of tuberin-deficient oncogenesis.

In the kidney, enhanced ROS levels have been consistently observed and well investigated in different pathophysiological conditions, such as ischemia-reperfusion, renal hypertension, chronic/acute renal failure and diabetic nephropathy^34–36^. Augmented ROS in renal proximal tubular epithelial cells, which represent more than 90% of the renal cortex contribute to the renal pathophysiological processes. Furthermore, the proximal tubular epithelial cells are sensitive to have mutations in tuberin gene to cause the pathology of renal angiomyolipomas observed in TSC patients. We found significantly increased levels of ROS in the renal angiomyolipomas obtained from patients with TSC. These results were confirmed in renal cortex of tuberin heterozygous mice. Furthermore, downregulation of tuberin in the renal proximal tubular epithelial cells enhanced the production of ROS, indicating that tuberin deficiency launches a signaling program that leads to increased ROS generation. Recently it has been shown that loss of *TSC1*, which codes for hamartin, in hematopoietic stem cells released them from quiescence to increased cell cycling due to increased production of ROS^37^. Similarly, *TSC1* deficiency in T lymphocytes resulted in increased ROS production, leading to apoptosis of these cells^38^. Our results in the present study demonstrate that tuberin deficiency causes increased production of ROS in renal proximal tubular epithelial cells. These results are consistent with the findings observed in the hematopoietic cells and demonstrate that disruption of TSC complex causes generation of ROS, which may contribute to the renal pathology found in TSC patients.

Many enzymes have been shown to contribute to the generation of ROS, such as NADPH oxidases, xanthine oxidases, lipoxygenases, heme oxygenases, and more importantly complexes in the electron transport chain of mitochondria during cellular respiration^39^. In the kidney, NADPH oxidases are the major sources of ROS^18,19^. Among many Nox enzymes, we identified Nox4 as the predominant isoform expressed in the proximal tubular epithelial cells. Our results show that tuberin downregulation increases the expression of the Nox4 protein. Surprisingly, the Nox4 mRNA expression was not affected. These results provide the first evidence that tuberin deficiency regulates Nox4 expression using a translational mechanism. Our results also conclusively demonstrate that tuberin downregulation induces the generation of ROS from Nox4 in the proximal tubular epithelial cells. These results are further confirmed by our observation that reconstitution of tuberin expression inhibited the expression of Nox4 in proximal tubular epithelial cells with downregulated tuberin.

Mutation of *TSC1* or *TSC2* gene or deficiency of any one of their protein products disrupts the TSC complex, which results in relieving the brake on mTORC1 activity^32^. In fact, renal angiomyolipomas from patients with TSC showed increased phosphorylation of S6 kinase due to activation of mTORC1^31^. This observation was mimicked in the tuberin-deficient cells in culture^22^. Similarly, enhanced mTOR activity has been reported in cancer cell proliferation^32^. Our results demonstrate that activated mTORC1 in tuberin-downregulated proximal tubular epithelial cells acts as the principal driver for increased Nox4 expression and ROS production. These results indicate that inhibition of mTORC1 may provide an attractive therapeutic choice for TSC. However, use of mTORC1 inhibitor in human cancers showed modest efficacy plausibly due to release of the feedback inhibition of the PI 3 kinase/Akt pathway similar to that found in *Tsc2* null cells^22,40–42^. Thus, alternative therapeutic modality may be useful to treat tuberin deficiency syndrome.

Nox-derived ROS has been suggested to play a critical role in cancer. ROS can interfere with redox-sensitive signaling pathways in cancer directly or indirectly, resulting in damage to lipid bilayers and alteration of post-translational modification, leading to autonomous cell growth^14^. Particularly in kidney, our group has established that oxidative stress, mediated by the NADPH oxidases of the Nox family, play a key role in VHL-deficient renal carcinogenesis^43^. While RCC was reported as rare in humans with TSC, more recent evidence using meta-analysis indicates that the frequency of RCC is much higher in these individuals than previously reported^8^. ROS can cause DNA damage, the major force driving the transformation of normal cells to cancer cells; however, the role of ROS in TSC or VHL LOH during the development of RCC is unknown and of great interests for the future research. Our group showed that tuberin inactivation reduces the expression of DNA repair protein OGG1 through Nox4^15^. This could be one of the potential mechanisms for explaining the correlation between loss of tuberin, enhanced Nox4 expression, and renal tumor growth.

In the current study, we have identified Nox4 as a source for ROS production in proximal tubular epithelial cells and the tuberin heterozygous mice. Although only 10% of these heterozygous mice develop angiosarcomas and renal carcinoma, DNA analysis reveals loss of another copy of Tsc2 allele in about 30% of lesions^11,12^. In the present study, we identified clear cystadenoma on the surface of most kidneys derived from *Tsc2*^+/−^ mice at the age of 1 year, along with prominent epithelial cell proliferation. Consistent with our *in vitro* data, we confirmed our observation of increased Nox4 protein expression and augmented ROS levels in the kidneys of *Tsc2*^+/−^ mice and renal angiomyolipomas obtained from patients with TSC. These results prompted us to test the Nox4 as a target for treating tuberin deficiency-derived tumors. Interestingly, use of Nox4-specific anti-sense oligonucleotide significantly prevented the tumor growth in nude mouse xenograft model, indicating the potential for targeting this molecule. Thus employing a specific inhibitor of Nox4, GKT, showed a significant reduction in the tumor burden in the xenograft model by inhibiting tumor cell proliferation. Furthermore, GKT delayed the initiation of tumor formation. These results conclusively demonstrate the efficacy of GKT in tuberin deficiency-generated tumorigenesis. It is important to note that pharmacokinetics of GKT has been worked out in humans^44^, and GKT is being used in a trial using patients with type 2 diabetes with albuminuria. In summary, our results provide a strong basis for the use of GKT in patients with TSC exhibiting renal angiomyolipomas and GKT may prove beneficial in the management of TSC-related pathologies.

## Methods

### Cell culture

Human renal proximal tubular epithelial cells were purchased from Lonza (Walkersville, MD), and cultured as indicated by the manufacturer. Rat epithelial cells RE3K and LEF2 cells were obtained from ATCC, and cultured in standard DMEM medium with 10% FBS. The tubular epithelial cells deficient in *TSC2* were established by infecting parental cells with *TSC2* shRNA lentiviruses in presence of polybrene (8 μg/ml), selected by 5% puromycin and maintained in standard DMEM medium with 10% FBS (Invitrogen).

### Tissue samples

Kidney angiomyolipoma tissues from TSC patients with angiomyolipoma and normal human kidney tissues were obtained from NICHD Brain and Tissue Bank for Developmental Disorders at the University of Maryland School of Medicine in accordance with a protocol approved by the UT Health San Antonio Institutional Review Board and Committee on Human Research.

### Plasmids, transfection, and infection

Control siRNA (sc-37007) and pools of siRNAs against tuberin (sc-36762) and Raptor (sc-44069), were purchased from Santa Cruz Biotechnology (Santa Cruz, CA); siRNAs against S6K1 (#SR304164) were from Origene Technologies, Inc. (Rockville, MD). siRNA transfection was performed using Oligofectamine reagent (Invitrogen) with OPTI-MEM serum-free medium (Invitrogen) according to manufacturer’s instructions. Suppression of protein expression was analyzed by immunoblotting. Lentiviral pLKO.1-puro vectors encoding shRNA specific for *TSC2* (#15478) or control scramble (#1864) were purchased from Addgene (Cambridge, MA). For lentiviral production, HEK 293 T cells in 10 cm dishes were transfected with 10 μg pLKO vectors together with packaging plasmids using Lipofectamine 2000 (Invitrogen) according to the manufacturer’s instruction. Culture growth media containing lentiviral particles were collected 48 and 72 hours post-transfection and filtered through 0.45 μm filters. Viral particles were pooled and stored at −80 °C. Two Raptor shRNA plasmids were described previously^45^.

### Construction of Nox4 UTR-Luc reporter plasmid

The 5′UTR sequence was identified in the UTRdb. The DNA fragment spanning −1 to −239 upstream of ATG start codon of Nox4 was subcloned into the pLightSwitch luciferase reporter vector (Switchgear Genomics, Carlsbad, CA). The sequence identity of the DNA fragment was confirmed by sequencing at the UT Health San Antonio Sequencing Core facility.

### Immunoblotting and Antibodies

Cells or tissues were collected/homogenized and lysed on ice in RIPA buffer (25 mM Tris-HCl, pH 7.5, 150 mM NaCl, 1 mM EDTA, 1% NP-40 and 5% glycerol) with protease inhibitors (#88660SPCL, Thermo Fisher Scientific) and phosphatase inhibitor mix (sc-45044, Santa Cruz). The lysates were further broken down by sonication for 10 seconds, prior to centrifugation at 13, 000 g at 4 °C for 30 minutes. Total protein concentration was quantified by Bio-Rad protein assay (#500-0006, Bio-Rad Laboratories, Inc.). Equal amounts of protein were subjected to 4–12% SDS-PAGE gels. Immunoblotting was performed by probing with following antibodies: anti-Nox4 (sc-30141) and anti-tuberin (sc-893) from Santa Cruz; monoclonal anti-beta-actin (A-5441) from Sigma; anti-human tuberin (#3635 s), anti-S6 kinase (#2708 s), anti-phospho-S6 kinase (T389) (#9205 s), anti-mTOR (#2983) were obtained from Cell Signaling Tech (Beverly, MA). The full scans of all blots are included in Supplementary Figures S5–S8.

### RNA Extraction and RT-PCR Analyses

Total RNA from cells or tissues was isolated by using PureLink^TM^ RNA mini kit (Ambion). cDNA reverse transcription was performed with High Capacity cDNA Reverse Transcription kit (Applied Bio System) and the amplified product was separated by agarose gel electrophoresis. Sequences of primers used are synthesized by IDT DNA Inc. and listed in Supplementary Table 1. Quantitative RT-PCR was performed with TaqMan PCR Master Mix (Applied Bio System) on Eppendorf Realplex Real-Time PCR System. The TaqMan probes are listed in Supplementary Table 1. Data were normalized to β-actin mRNA levels.

### Measurement of Oxidative stress by DHE (dihydroethidium) staining, H_2_-DCFDA (2′,7′-dichlorodihydrofluorescein diacetate) flow cytometry and Amplex red assay

For DHE staining, Human proximal tubular epithelial cells were seeded in 4-chamber slides. After 48–72 hours of siRNA transfection, cells were briefly washed with PBS and incubated in 1 μM DHE (#D1168 Thermo Fisher) for 30 minutes. Then, cells were quickly washed with PBS for 3 times, fixed in 4% paraformaldehyde for 30 min at room temperature. After fixation, cells were washed again for 3 times and immediately subjected to confocal microscopy imaging. Representative fluorescent images from random regions were taken, and the fluorescence signals of DHE staining area were determined using ImageJ software. Cellular oxidative stress was also measured by H_2_-DCFDA dye (#D399, Thermo Fisher). Briefly, the medium was replaced with DMEM containing 1 μM H_2_-DCFDA dye for 30 minutes in dark. Then, cells were washed with PBS, collected by 0.25% trypsin solution, fixed in cold 75% ethanol, and stored at −20 °C for 16 hours. Fixed cells were subsequently quantified in a BD Biosciences FACS Calibur (UTHSCSA Flow Cytometry Core) with 10,000 events for total cell population using BD Biosciences Cell Quest software, and the data were analyzed by FlowJo software (Ashland, OR).

To measure the hydrogen peroxide in tissue, we used Amplex Red Hydrogen Peroxide Assay Kit (A12222, Thermo Fisher) and performed the experiments according to the vendor’s protocol. 100 μg of lysates in 100 μl of Kreb’s ringer buffer was used and assay was initiated by adding 100 μl of the same buffer containing 50 μM Amplex red together with 0.1 U/ml horseradish peroxidase. Instant formation of resorufin fluorescence was measured using an EnSpire multimode plate reader at excitation and emission wavelengths 540 nm and 595 nm, respectively. The data were normalized with total protein input and averaged from at least 5 repeats.

### Measurement of NADPH oxidase activity by lucigenin chemiluminescence assay

NADPH oxidase activity was measured in cells grown in serum-free medium or in kidney cortex isolated from human or mice as previously described^29^. Cultured cells were washed five times with ice-cold PBS and scraped from the plates followed by centrifugation at 800 rpm, 4 °C, for 10 min. The cell pellets were then re-suspended in lysis buffer (20 mM KH_2_PO4, pH 7.0, 1 mM EGTA, with proteinase inhibitors). Cell suspensions or washed kidney cortex were homogenized with 100 strokes in a Dounce homogenizer on ice. To start the assay, 20 μg of homogenates were added to assay buffer (50 mM phosphate buffer, pH 7.0, containing 1 mM EGTA, 150 mM sucrose) with 5 μM lucigenin and 100 μM NADPH. Photon emission were measured as relative light units every 30 seconds for 5 minutes in a luminometer. A buffer blank (<5% of the cell signal) was used as a control from each reading. Superoxide production was expressed as relative light units per milligrams of protein.

### Xenograft model of *Tsc2*-null tumor and treatments

4 weeks old male Nu/Nu mice were obtained from Harlan Laboratories (Indianapolis, IN) and fed on regular chow for 2 weeks upon arrival. At the age of 6 weeks, animals were injected with 3 × 10^6^ LEF2 cells subcutaneously in the flank region. Animals were monitored for tumor growth for 7–8 weeks, and the tumors were measured with a caliper twice a week. At the end of experiments, mice were sacrificed; tumors were collected for analyses. All work related to animals was by the protocol approved by the Institutional Animal Care and Use Committee at The University of Texas Health San Antonio.

Antisense oligonucleotides were designed to suppress the expression of rat Nox4 at the ATG start codon (5′-AGCTCCTCCAGGACAGCGCC-3′). Antisense and corresponding sense oligo or PBS were administered subcutaneously 24 hours after implantation of *Tsc2* null cells in mice, for up to 8 weeks, at a dosage of 45 mg/kg body weight/day. GKT137831 (GKT), an inhibitor for Nox1/4, was obtained from Genkyotex Inc (Switzerland). GKT was mixed with standard chow diet and treatment with a dosage of 40 mg/kg/day was started at the next day after administration of *Tsc2* null cells in mice.

### Immunohistochemical Immunofluorescence staining

Localization of PCNA (1:200, Abcam) was assessed in paraffin-embedded sections (5 μm thick) by alkaline phosphatase histochemistry. Representative images from random regions were taken and the intensities of signals were determined using ImageJ software.

### Statistical analysis

Data are presented as means ± SE. Statistical analysis between multiple groups was performed by one-way ANOVA (nonparametric), and post-test analysis was performed using Tukey Statistical by GraphPad Prism software. A *P* value of 0.05 or less was considered statistically significant.

## Electronic supplementary material

Supplementary Figures
