# Supplementary material for: Nox4 is a Target for Tuberin Deficiency Syndrome
Source: Sci Rep. 2018 Feb 28;8:3781. doi: 10.1038/s41598-018-21838-4 (PMC5830489; doi:10.1038/s41598-018-21838-4)
Supplement: Supplementary file 1 — Supplementary Figures [file 41598_2018_21838_MOESM1_ESM.pdf]

Nox4 is a Target for Tuberin Deficiency Syndrome

Qian Shi[1,2*], Suryavathi Viswanadhapalli[1], William E. Friedrichs[1], Chakradhar Velagapudi[1], Cédric Szyndralewiez[5], Shweta Bansal[1], Manzoor A. Bhat[2], Goutam Ghosh Choudhury[1,3,4*] and Hanna E. Abboud[1,3§]

From the VA Biomedical Laboratory Research[3] and Geriatric Research, Education and Clinical Center[4], South Texas Veterans Health Care System, San Antonio, Texas; Departments of Medicine[1] and Cellular and Integrative Physiology[2], UT Health at San Antonio, Texas; Genkyotex SA, Geneva, Switzerland[5]

§ Deceased on January 7, 2015

Running title: *Tuberin deficiency upregulates Nox4*

*To whom correspondence should be addressed:

Qian Shi, Ph.D., Departments of Cellular and Integrative Physiology, UT Health San Antonio, Texas; Email: shiq@uthscsa.edu

and Goutam Ghosh Choudhury, Ph.D., Department of Medicine, UT Health San Antonio, Texas; Email: choudhuryg@uthscsa.edu

Keywords: Tuberin, NADPH oxidase, ROS, renal epithelial cells, mTOR

Supplementary Table-1.
Primers and Taqman probes for Nox protein family and internal control.

Genes	Primers for SYBR assay	Cat# of Taqman probes
Nox1	For: TTGTTTGGTTAGGGCTGAATGT<br>Rev: GCCAATGTTGACCCAAGGATTTT	Hs00246589_m1
Nox2 (CYBB)	For: AACGAATTGTACGTGGGCAGA<br>Rev: GAGGGTTTCCAGCAAACTGAG	Hs00166163_m1
Nox3	For: CGTGGCGCATTTCTTCAACC<br>Rev: GCTCTCGTTAGGGGTGTTGC	Hs01098883_m1
Nox4	For: TTGGGGCTAGGATTGTGTCTA<br>Rev: GAGTGTTCGGCACATGGGTA	Hs00418356_m1
Nox5	For: GTGACTCAGCAGTTTAAGACCAT<br>Rev: GGACTCTTTCACATGCAGAGC	Hs00225846_m1
β -Actin	For: AGGCACCAGGGCGTGAT<br>Rev: GCCCACATAGGAATCCTTCTGAC	Hs01060665_g1

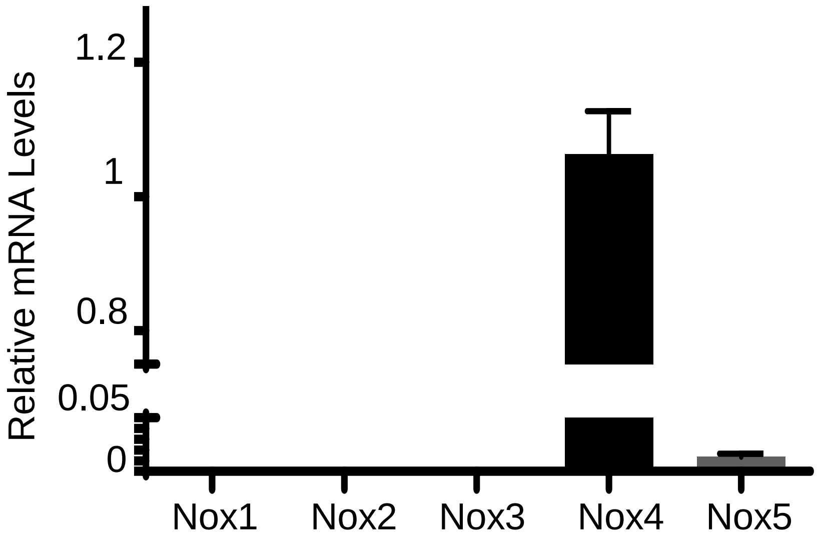

Supplementary Figure S1. Nox isoform mRNA levels were measured by quantitative RT-PCR in the proximal tubular epithelial cells, using the Taqman probes listed in Supplementary Table-1.

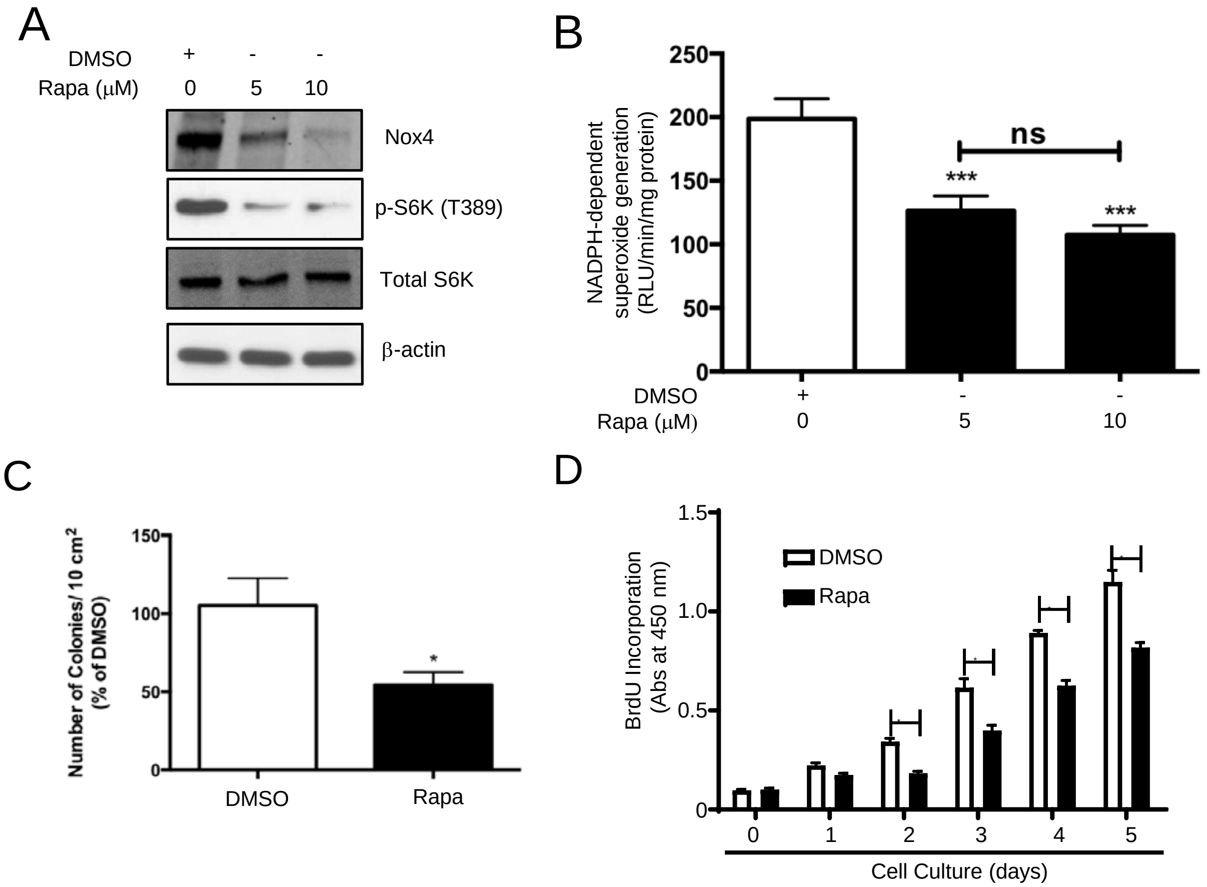

Supplementary Figure S2. Rapamycin significantly suppresses the expression and activity of Nox4, and the growth of tuberin-deficient LEF2 cells *in vitro.* (A-B) Tuberin-null LEF2 cells were incubated with rapamycin as indicated. The cell lysates were immunoblotted with indicated antibodies (A) and assayed for NADPH oxidase activity using lucigenin chemiluminescence assay (B). (C) Colony formation was inhibited by Rapamycin (10 μM) in LEF2 cells; the measurement was performed at the 7th day of treatment. **p < 0.01 between DMSO and Rapamycin groups. (D) BrdU incorporation assay was performed in LEF2 cells with Rapamycin (10 μM) for continuous 5 days, and absorbance was measured at 450 nm each day;

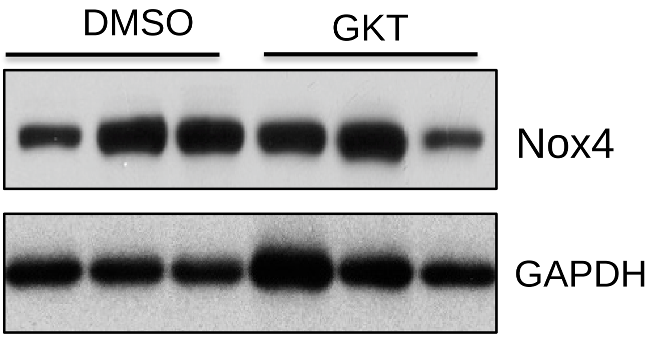

Supplementary Figure S3. GKT does not have any effect on Nox4 protein levels. Nox4 and GAPDH protein expression were measured by western blot in LEF2 cells treated with GKT (10 μM) or DMSO.

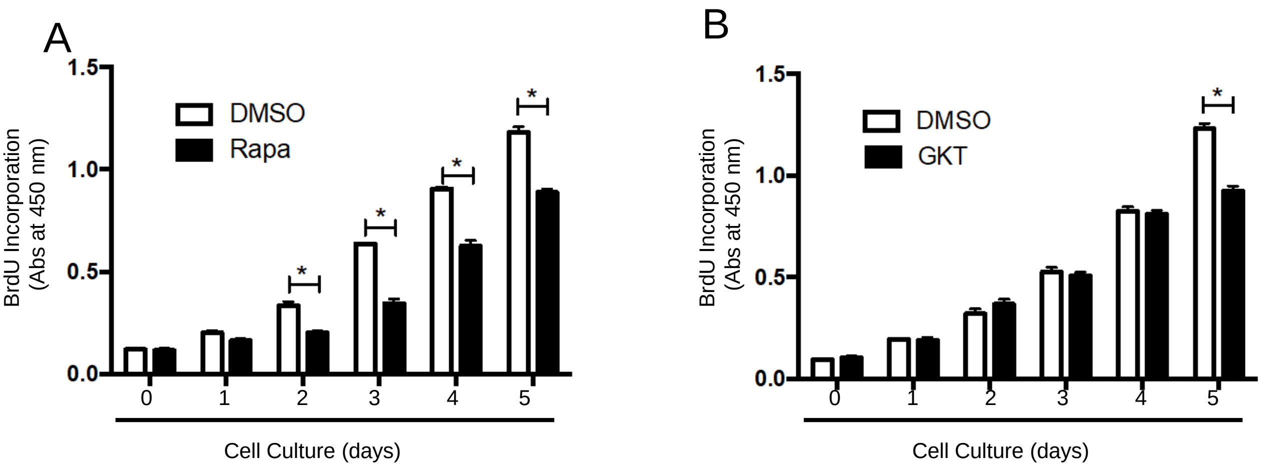

Supplementary Figure S4. Rapamycin and GKT significantly suppress the growth of tuberin deficient cells *in vitro.* BrdU incorporation assay was performed in shTSC2 cells in the presence of Rapamycin (10 μM) (A) and GKT (10 μM) (B) at the indicated times. *p < 0.05, *n*=5, between DMSO and Rapamycin or GKT groups.

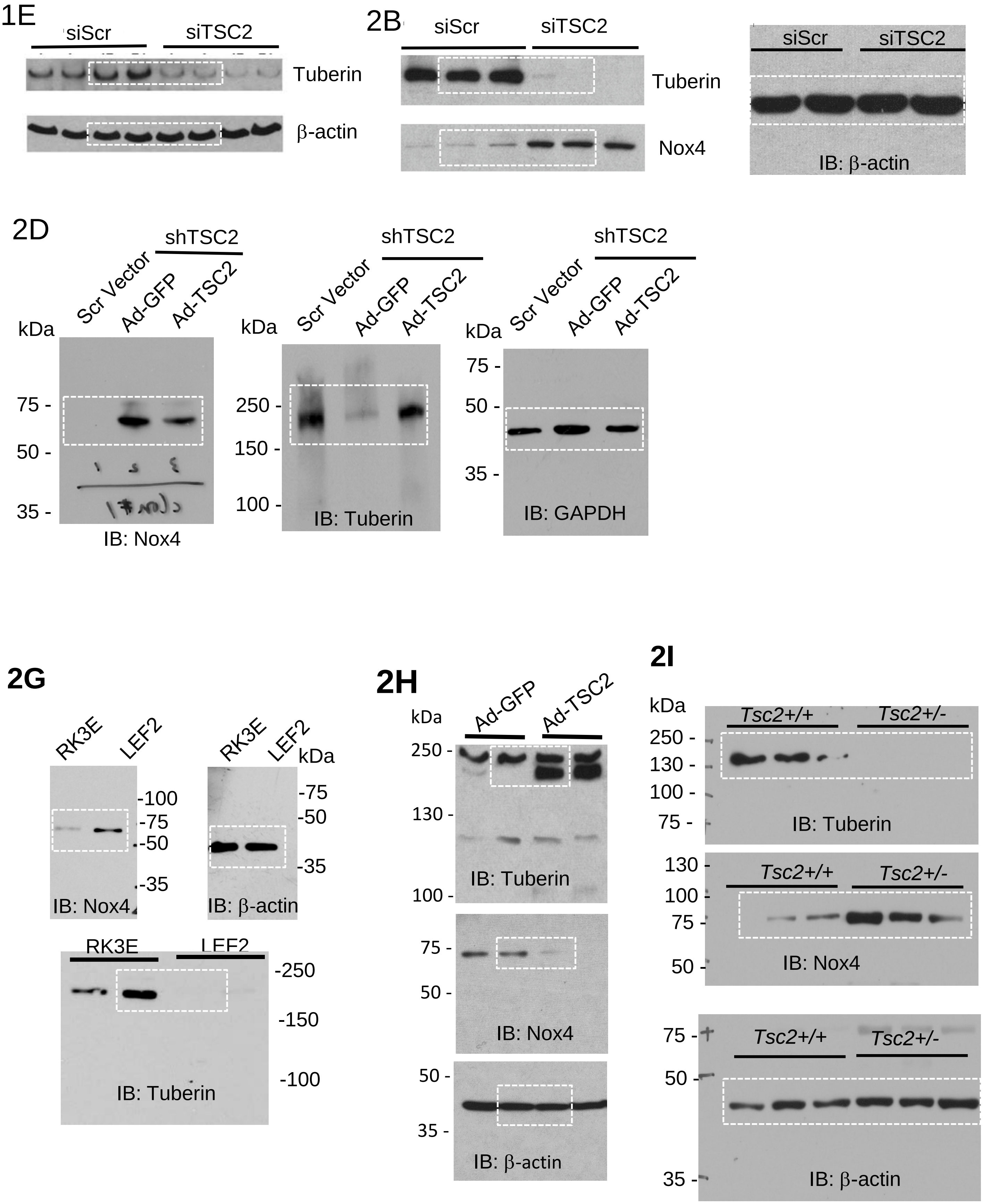

Supplementary Figure S5. Full western blot scans for Figures 1-2.

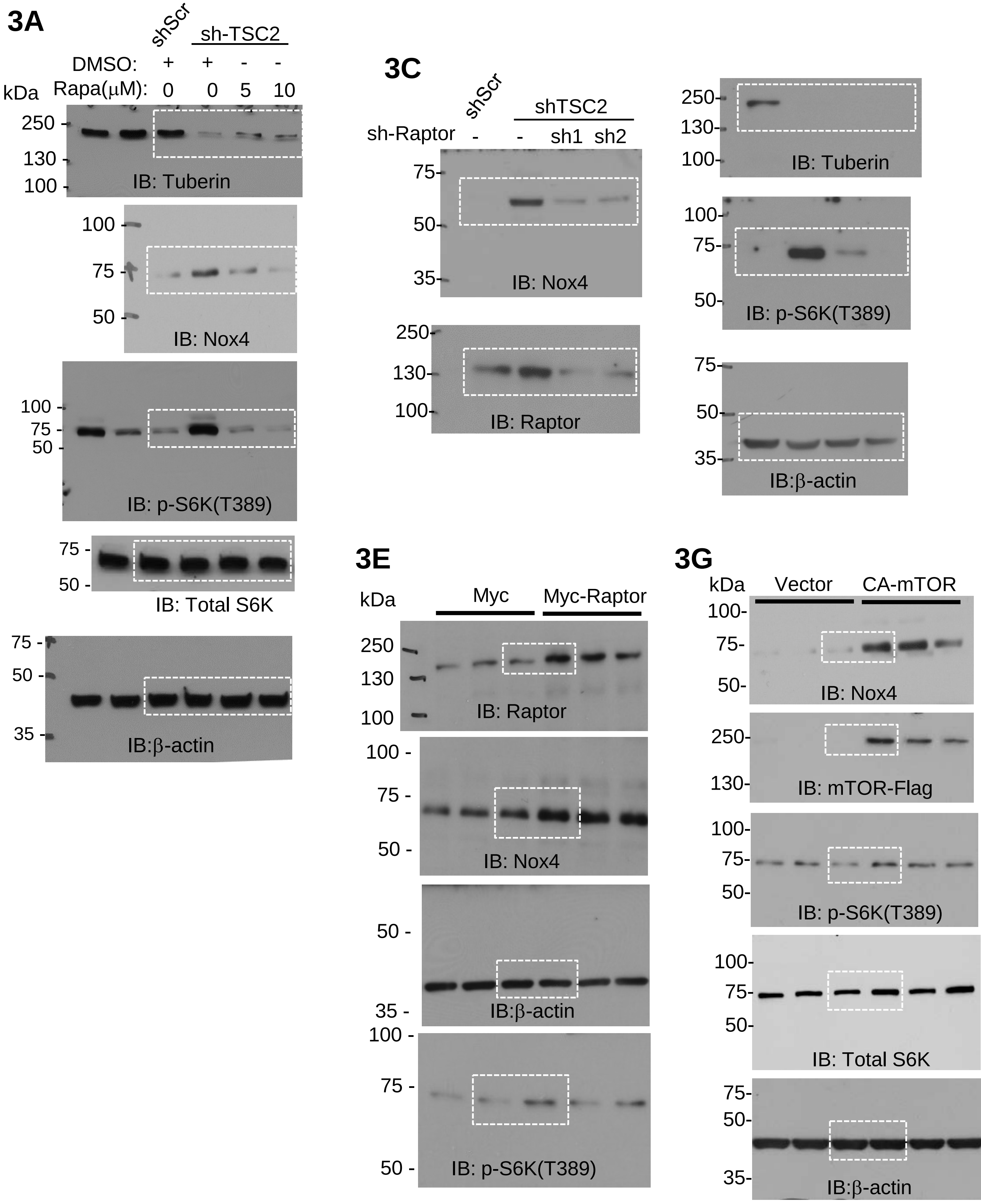

Supplementary Figure S6. Full western blot scans for Figure 3.

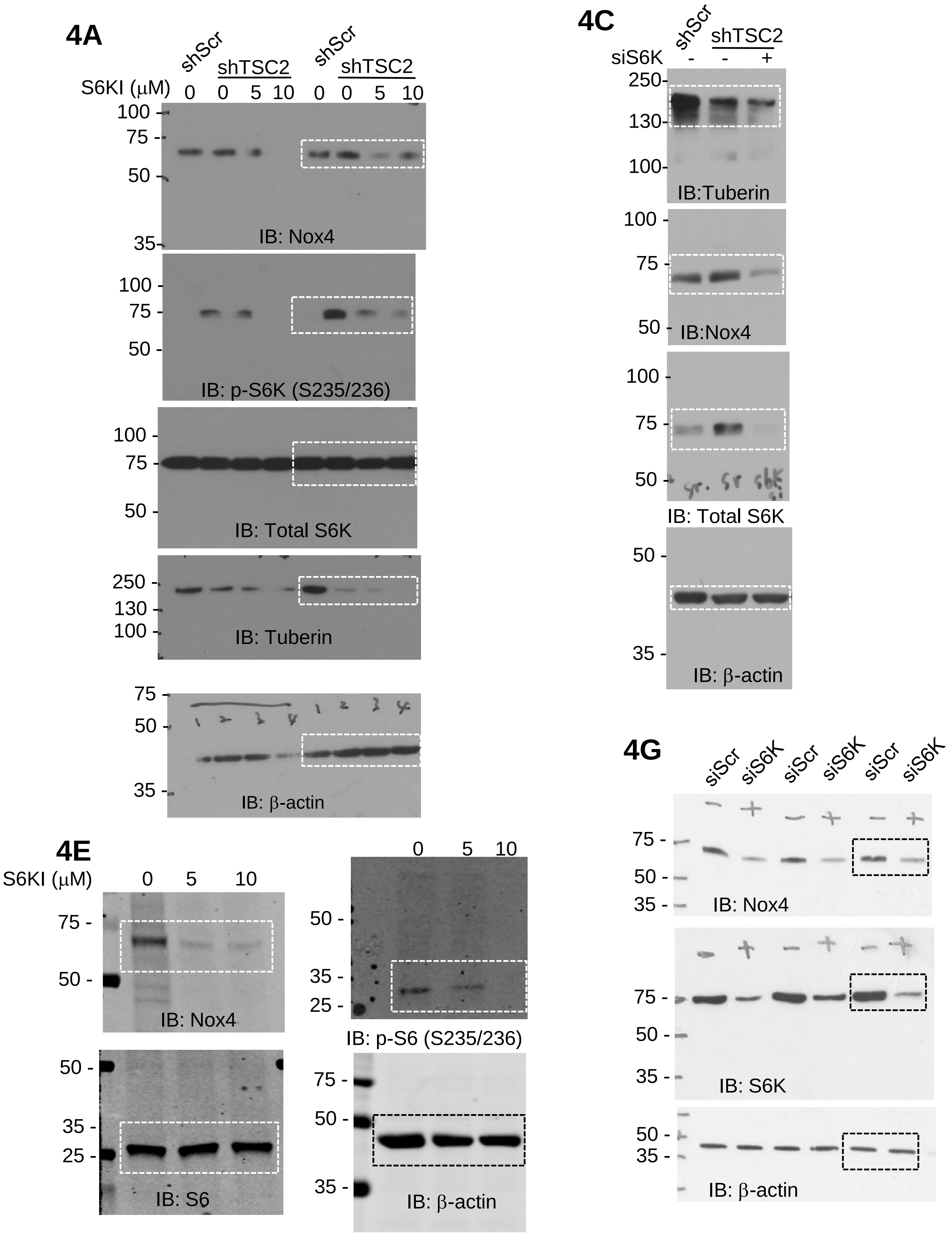

Supplementary Figure S7. Full western blot scans for Figure 4.

5A

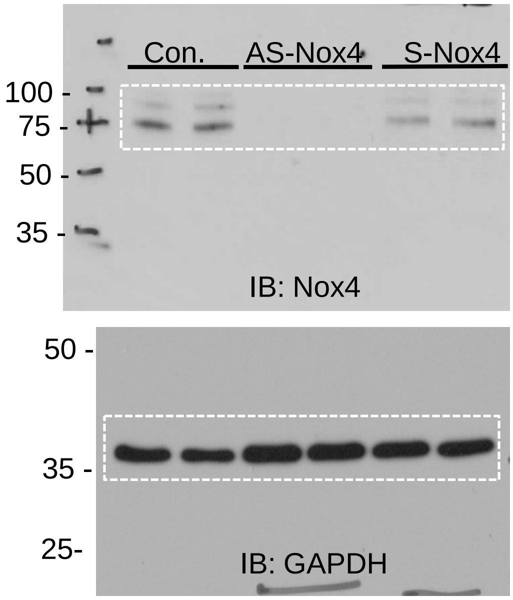

5E

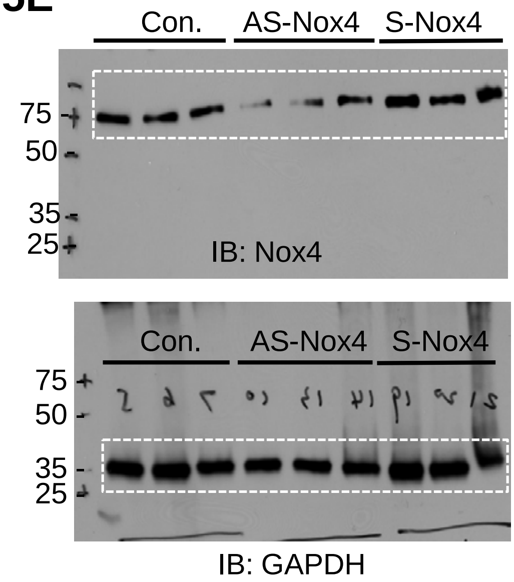

S2-A

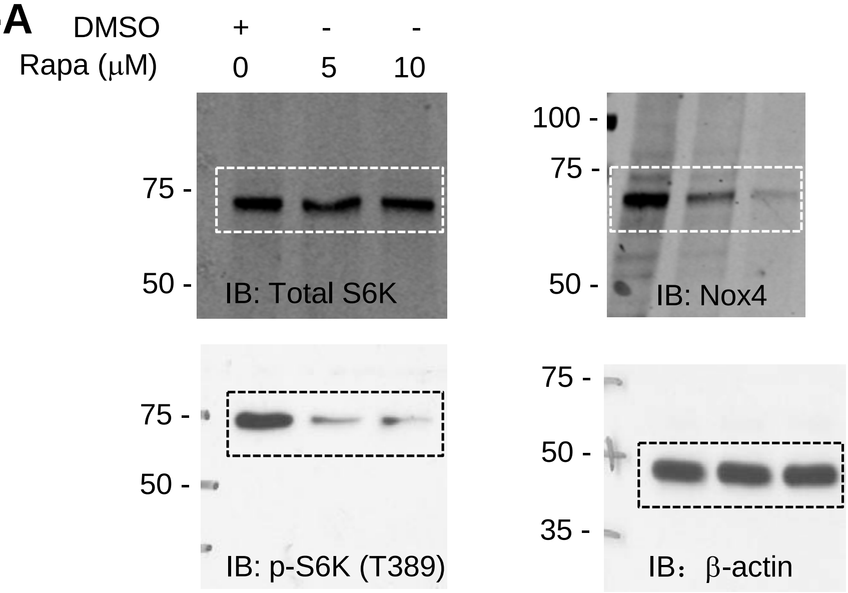

S3

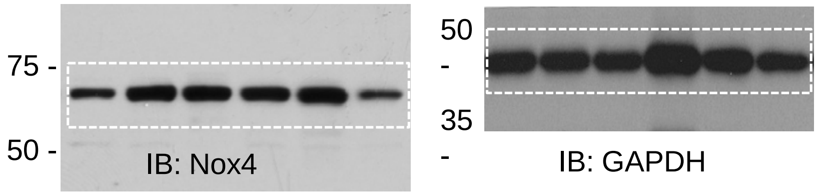

Supplementary Figure S8. Full western blot scans for Figure 5, Supplementary Figures S2 and S3.
